# Empowering Sustainable Growth and Workforce: Unveiling Challenges and Strategies for Retaining Community Pharmacists in Malaysia

**DOI:** 10.3390/pharmacy11050163

**Published:** 2023-10-12

**Authors:** Khim Lynn Ooi, Kingston Rajiah, Mari Kannan Maharajan, Pe Sei Wong

**Affiliations:** 1 School of Postgraduate Studies, International Medical University, Kuala Lumpur 57200, Malaysia; khimlynn91@gmail.com; 2 School of Pharmacy and Pharmaceutical Sciences, Ulster University, Coleraine BT52 1SA, UK; 3 School of Pharmacy, University of Nottingham Malaysia, Selangor 43500, Malaysia; marikannan.maharajan@nottingham.edu.my; 4 School of Pharmacy, International Medical University, Kuala Lumpur 57200, Malaysia; peise_wong@imu.edu.my

**Keywords:** community pharmacists, job retention, workforce management, work–life balance, value and trust

## Abstract

Background: Community pharmacists face challenges in job retention due to compensation, work–life balance, and career growth concerns. With pharmacists’ evolving roles in healthcare, retaining them becomes crucial for maintaining quality service. Addressing their needs is vital for a skilled healthcare workforce. This study evaluates job retention among community pharmacists, considering various workforce management domains and demographic characteristics. Materials and Methods: A cross-sectional study was employed with a self-administered questionnaire among community pharmacists in Kuala Lumpur and Selangor, Malaysia. Spearman’s correlations and ordinal logistic regression analysed job retention relationships with workforce domains and predicted the demographic characteristics. Results: A total of 414 participants attempted the survey, of which 311 completed the study. Strong correlations linked job retention with value, trust, and work–life balance. Female pharmacists had higher retention odds, while younger pharmacists had lower retention odds. Pharmacists with over 10 years of experience showed higher retention odds. Discussion: ‘Value and trust’ and ‘work–life balance’ were pivotal for the job retention of community pharmacists. Strategies boosting value, trust, and work–life balance was vital. Gender, age, and experience also predicted job retention. Conclusions: Cultivating trust, valuing contributions, and providing a work–life balance can enhance job retention and commitment.

## 1. Introduction

Community pharmacists face job retention challenges influenced by factors such as compensation, work–life balance, and career growth [1]. The evolving role of pharmacists in delivering healthcare further highlights the importance of retaining these professionals. As the demand for pharmacists grows, addressing their unique needs becomes crucial for maintaining a skilled and motivated workforce in the dynamic healthcare landscape. Job retention involves keeping employees within an organisation, contributing to its objectives and competitiveness [2]. Achieving this requires understanding individual employees’ needs, as not all employees share the same values and attitudes. Retaining competent employees is crucial because of the costs associated with human resources, a loss of skills, and team dynamics [3]. Employee retention strategies include transparent selection processes, fair compensation, recognition programs, growth opportunities, and open communication [4].

Numerous factors may influence employee job retention, including compensation [5], work–life balance [6], work environment [7], training and leadership [8], management support [9], and promotional opportunities [10]. Identifying these factors allows the management to implement effective strategies. Failing to retain employees leads to a loss of skills, knowledge, and team dynamics, impacting a company’s competitiveness [11]. A report from Curado et al., 2022, mentioned that the key retention factors of workforce management domains include management care, communication, value and trust, management effectiveness, career development, and work–life balance [12].

Management care involves considering employee well-being and career growth [13]. Communication provides a clear role for expectations and prevents employee frustration [14]. Values and trust are built through open communication and participation in decision-making [15]. Management effectiveness concerns timely support and leadership during these challenges [16]. Career development opportunities tailored to individual needs enhance retention [17]. Work–life balance through flexible schedules promotes employee job retention [18].

As pharmacists’ roles expand, managing their work environment becomes crucial for quality healthcare. Workforce management involves efficient human resource practices, with leadership from management, line managers, and employees [19]. Investigating these domains among community pharmacists can help guide management efforts. Also, tailored strategies, ongoing communication, and feedback are essential for employee retention. Retaining employees also benefit this organisation by reducing turnover, improving performance, and ensuring quality healthcare delivery.

In Malaysia, community pharmacists face significant job retention challenges that arise from a combination of factors, such as demanding workloads, long working hours, and increasing responsibilities, often leading to burnout and decreased job satisfaction among these professionals [20,21,22,23]. Moreover, the absence of well-defined career progression pathways within the field may discourage them from staying in their long-term roles. Work–life balance is another critical concern. Extended hours and irregular schedules in community pharmacy settings can strain personal and family life, which might contribute to overall dissatisfaction. By recognising and addressing these issues, the healthcare industry can better retain its valuable pharmacy workforce and ensure consistent and high-quality patient care.

As job retention is essential for an organisation’s success and competitiveness, this study evaluates the associations between community pharmacists’ job retention and pharmacy workforce management domains. In addition, this study determines predictive factors for community pharmacists’ job retention.

## 2. Materials and Methods

### 2.1. Ethics Approval

This study was approved by the International Medical University (IMU) Joint Committee on Research and Ethics, Kuala Lumpur, Malaysia (Project ID: MPP I/2020(09)).

### 2.2. Study Setting, Sampling, and Participants’ Recruitment

A cross-sectional study was conducted using a self-administered questionnaire distributed to community pharmacists working in the states of Kuala Lumpur and Selangor in Malaysia from January 2021 to June 2021. Data were collected through an online survey [24]. Convenience sampling was conducted by distributing the questionnaire online to community pharmacy networks through verified social media groups of community pharmacists and community pharmacies’ official email. In addition, snowball sampling [25] was performed on pharmacists who responded to the survey. Informed consent was obtained before participation in the study. An explanation of the study was provided to participants on the first page of the online questionnaire. The participants could either continue to the next page or exit on the first page after reading the explanation. The confidentiality of participants’ data was also addressed, such that data obtained from this questionnaire is strictly for the concerned study only. Participants’ eligibility criteria were fully registered pharmacists working in community pharmacies of the Kuala Lumpur and Selangor states. Any number of pharmacists from the same pharmacy store could participate in the study. Each participant could fill out the questionnaire only once. To ensure that participants met the eligibility criteria, they had to fill in the state where they were employed, the name of the pharmacy they were working in, their pharmacy location, and their mobile number to identify a single entry.

### 2.3. Sample Size

The sample size was calculated using a Raosoft calculator with a 5% margin of error and a 95% confidence interval. Approximately 945 community pharmacies were present in Kuala Lumpur and Selangor states as of August 2020 [26]. At least 274 community pharmacists were required to participate in this study. A total of 311 community pharmacists completed this study out of the 414 participants, which was greater than the required sample size.

### 2.4. Study Questionnaire

The online questionnaire was adapted and modified from resources relating to job retention and workforce management [27,28]. The questionnaire was provided as Supplementary Materials. The questionnaire consisted of 47 items and comprised sections A, B, and C: Section A: participants’ demographics (7 items), Section B: job retention (10 items), and Section C: workforce management domains (6 domains, 30 items) (management care-5 items, management communication-5 items, value, and trust-5 items, effectiveness-5 items, career development-5 items, work–life balance-5 items). In sections B and C, each item had a five-point Likert scale ranging from ‘strongly disagree’ (lowest score 1) to ‘strongly agree’ (highest score 5) to determine pharmacists’ opinions on job retention and workforce management. In this study, ‘management care’ refers to the well-being, growth, and support of employees, fostering a workplace environment that values and attends to their needs, both personal and professional. ‘Management communication’ refers to the strategies and processes through which leaders convey information, expectations, and organisational goals to employees, promoting transparency, alignment, and effective collaboration. ‘Value and trust’ refers to individuals feeling respected and appreciated for their contributions and trusting their colleagues and leadership to act with integrity and reliability. ‘Management effectiveness’ refers to the ability of leaders to achieve organisational objectives efficiently while also nurturing employee satisfaction, development, and engagement. ‘Career development’ refers to the planning and actions taken by individuals and organisations to enhance an individual’s skills, knowledge, and experiences, aligning them with their career goals and the needs of the organisation. ‘Work–life balance’ refers to the equilibrium between one’s professional responsibilities and personal life, ensuring that the demands of work do not overwhelm or encroach upon personal well-being and fulfilment.

### 2.5. Validity and Reliability of the Questionnaire

This questionnaire underwent content validation by ten community pharmacists, followed by face validation performed by three experts, a psychologist, human resource personnel, and a pharmacist. The inputs received are included in the questionnaire. The questionnaire was also piloted on twenty community pharmacists. Cronbach’s alpha test was conducted for internal reliability using the Statistical Package for the Social Sciences (SPSS) version 24. Cronbach’s alpha scores for sections B and C were 0.783 and 0.798, respectively.

### 2.6. Data Analysis

Frequencies and percentages were applied to demographic data. Tests for the normality of data were conducted by visually examining each histogram plot and conducting the Kolmogorov–Smirnov and Shapiro–Wilk tests. As visual analyses and normality tests showed a significant skew, non-parametric tests were used in this study. As the distribution of data was not normal, the median and interquartile range (IQR) were used. The cutoff point threshold was calculated as 50% based on the cutoff point formula proposed by Barua (2013) [29]. The defined threshold limit of 50% establishes two possible outcomes for job retention and workforce management: ‘positive’ (when the median falls between 2.5 and 5) and ‘negative’ (when the median ranges from 1 to 2.4). To illustrate, if the total mean score was 50% or higher, it indicated positive job retention (indicating participants’ interest in retaining their jobs), whereas scores below 50% signified negative job retention (suggesting that participants were not interested in retaining their jobs). Spearman’s multiple correlations test was used to determine the strength of the correlation between community pharmacists’ job retention and workforce management domains with a *p*-value < 0.05, which was measured at a 95% confidence level. Outliers were investigated using a Mahalanobis distance of 7.82, which was within the limit [30]. Cohen’s correlation coefficient was evaluated to determine the strength of the effect size; correlation coefficients from 0.10 to 0.29 represented a weak correlation, coefficients from 0.30 to 0.49 represented a moderate correlation, and coefficients of 0.50 and greater represented a strong correlation. Ordinal logistic regression was performed to determine the demographic characteristics that predicted pharmacists’ job retention.

## 3. Results

A total of 414 participants attempted the survey, of which 311 completed the study with a response rate of 75.12%. The demographic characteristics of the participants are presented in Table 1. Most of the participants were female (n = 224, 72.03%). Most participants were early-career pharmacists aged between 21 and 30 years (n = 231, 74.28%). The majority of the participants were of Chinese ethnicity (n = 263, 84.57%). In terms of work experience, most participants had three years or less of experience (n = 213, 68.49%). The majority of participants were from chain pharmacies (n = 250, 80.39%). In this study, we found that the majority of community pharmacies had between two and five pharmacists as staff. Specifically, out of the total sample size of community pharmacies (n = 234), approximately 75.24% (n = 176) of the pharmacies had two or more pharmacists.

The descriptive analysis results of job retention and workforce management domains, “positive” or “negative”, are summarised in Table 2. The average median score for pharmacists’ job retention was four (IQR = 4–5). Approximately 70% of the participants responded positively to job retention (“Agree” = 56.17% and “Strongly Agree” = 13.73%). The average median score for management care was three (IQR = 3–4). About 65% of the participants responded positively to management care (“Agree” =51.96% and “Strongly Agree” = 12.48%). The average median score for management communication was three (IQR = 3–4). About 62% of the participants gave positive responses for management communication (“Agree” = 53.12% and “Strongly Agree” = 8.49%). The average median score for value and trust was two (IQR = 2–3). About 57% of the participants gave negative responses for value and trust (“Disagree” = 51.06% and “Strongly disagree” = 5.92%). The average median score for management effectiveness was three (IQR = 3–5). About 72% of the participants responded positively to management effectiveness (“Agree” = 49.97% and “Strongly Agree” = 21.54%). The average median score for career development was three (IQR = 3–5). Approximately 52% of the participants responded positively to career development (“Agree” = 42.32% and “Strongly Agree” = 9.26%). The average median score of work–life balance was two (IQR = 2–3). About 53% of the participants responded negatively to work–life balance (“Disagree” = 44.76% and “Strongly disagree” = 8.75%).

The correlation results for job retention in the workforce management domains are presented in Table 3. There was a weak positive correlation between management care and job retention (r = 0.29, *p* = 0.034). There was a weak positive correlation between management communication and job retention (r = 0.28, *p* = 0.037). There was a strong positive correlation between value, trust, and job retention (r = 0.61, *p* = 0.003). There was a moderate positive correlation between effectiveness and job retention (r = 0.49, *p* = 0.015). There was a moderate positive correlation between career development and job retention (r = 0.42, *p* = 0.028). There was a strong positive correlation between work–life balance and job retention (r = 0.55, *p* = 0.001).

The ordinal logistic regression results for demographic characteristics that predicted job retention are presented in Table 4. The standardised beta estimate (B) represented the probability of independent variables falling into the category of the dependent variable. The odds ratio (OR) represented the odds of the dependent variable falling into the higher/lower category with the unit of change in the independent variable. In terms of gender, female pharmacists were statistically more likely to retain their jobs than male pharmacists (OR = 1.374, *p* = 0.045). In terms of age, younger pharmacists were statistically less likely to retain their jobs than their older counterparts (OR = 0.756, *p* = 0.021). In terms of work experience, experienced pharmacists were statistically more likely to retain their jobs than less experienced pharmacists (OR = 1.215, *p* = 0.002).

## 4. Discussion

This study emphasised the significance of various workforce management domains when influencing job retention among community pharmacists in Kuala Lumpur and Selangor states in Malaysia. While factors such as value and trust and work–life balance impacted job retention, demographics such as gender, age, and experience also played roles. As this study has unveiled challenges in retaining community pharmacists, various strategies have been discussed to enhance job satisfaction, employee commitment, and overall retention within the community pharmacy sector to empower sustainable growth and create a decent work atmosphere.

The results reveal that the majority of pharmacists provided positive responses, indicating agreement with their job retention. This positivity could be due to various factors, such as job security, growth opportunities, and job satisfaction, which have been identified as important contributors to employee retention in previous studies [5,6,7,8,9,10]. This might also be a favourable opinion among pharmacist towards their current employment, as fear of job loss can lead to favouring the workplace [31]. 

Management care and communication emerged as areas where pharmacists were positive. These findings reflect the significance of the management’s role in cultivating a supportive and engaging working environment. Pharmacists’ recognition of management’s care toward their well-being and career growth implied that efforts in these domains are being acknowledged and appreciated. Management communication, as evidenced by the positive responses, indicates that communication might enhance pharmacists’ understanding of their roles and responsibilities, fostering a sense of clarity and direction. Studies have also reported that management care and communication impact employee satisfaction [32,33].

However, the domains of value and trust are more challenging. A notable percentage of pharmacists expressed negative responses, indicating a lack of perceived transparency and openness between the management and employees. This highlights the importance of building mutual trust and creating an environment where employees feel valued and included in their decision-making processes. Organisations may need to invest effort into enhancing communication practices, acknowledging employee contributions, and involving them in shaping policies and initiatives. Studies have reported that job satisfaction depends on feeling valued for one’s contributions, and not feeling valued in the workplace can employees to leave their jobs [34,35], whereas trust in employees influences their workplace performance [36].

Work–life balance also demonstrated negative perceptions, with the majority of pharmacists expressing dissatisfaction. The demanding nature of the pharmacy profession, coupled with long working hours and irregular schedules, can lead to heightened stress and reduced job satisfaction. Pharmacists often find it challenging to balance their professional commitments with their personal responsibilities, which may include family, hobbies, and self-care. Consequently, this imbalance can lead to burnout, decreased motivation, and attrition. Promoting a culture of self-care and well-being within the organisation can have a positive impact on work–life balance [37]. Providing wellness programs, access to counselling services, and encouraging breaks throughout the workday can help pharmacists manage their stress and enhance their overall job satisfaction. These initiatives could convey that the organisation values its employees’ health and mental well-being, fostering a sense of loyalty and commitment [38,39].

On the positive side, management effectiveness and career development were moderately favoured. These results underscore the significance of these domains in influencing job retention among community pharmacists. The effectiveness of community pharmacy managers can ensure that work is completed efficiently and on time for pharmacists and, hence, provide positive opinions. Studies have shown that managers’ effectiveness increases employee satisfaction and performance at work [40,41]. Continuous professional development (CPD) in community pharmacies may have been a reason for this positivity among community pharmacists. In Malaysia, attending CPD events is essential to retain and renew pharmacist registration certificates. CPD events expose pharmacists to new knowledge, skills, and best practices, enabling them to remain updated with emerging trends and evidence-based approaches. Studies have also reported that employee growth through skill-building [42] enhances performance, morale, and engagement. Employers gain skilled staff, have higher satisfaction, and reduce turnover [43,44]. A strong positive correlation was observed between values, trust, and pharmacists’ job retention. This highlights the critical role of open communication and employee involvement in organisational decisions. When employees feel valued and trusted, their commitment to the organisation tends to strengthen [45]. The strong correlation between work–life balance and job retention further emphasises its crucial role. In an era where work-related stress and burnout are prevalent, offering a flexible work environment that accommodates personal responsibilities can significantly contribute to retaining valuable employees [46].

The finding that female pharmacists are more likely to retain their jobs than their male counterparts suggests potential gender-based differences in job retention and commitment. This is aligned with broader trends in the workforce, where women often seek workplaces that offer flexibility and a supportive environment [47]. Younger pharmacists, aged between 21 and 30 years, were found to have lower odds of job retention than their older counterparts. This could be attributed to the exploratory nature of their early career stages, where younger pharmacists might be more willing to explore different opportunities and gain diverse experiences. Studies have reported that younger generations tend to change their careers and try various roles [48,49]. By contrast, more experienced pharmacists (with over 10 years of experience) demonstrated higher odds of job retention. This aligns with the notion that employees tend to invest more in their roles as they accumulate experience, expertise, and a deeper understanding of their organisation’s dynamics. Studies have reported that employees with more experience have higher levels of engagement and are more likely to take an active role [50,51].

The findings of this study have important implications for the field of community pharmacies and beyond. Organisations can utilise these insights to fine-tune their workforce management strategies and enhance job retention rates. A holistic approach that addresses communication, career development, work–life balance, and managerial effectiveness could contribute to fostering a positive work environment that is conducive to employee commitment. Five key strategies can be followed to achieve this holistic approach. Firstly, transparent communication can be established to cultivate trust and value. Secondly, mentorship programs and skill development opportunities can be offered to ensure growth and work–life balance. Thirdly, implementing flexible work arrangements and recognising individual needs is important. Fourthly, encouraging open dialogue about goals and concerns can foster a sense of belonging. Lastly, to retain young employees, introducing recognition programs and involving them in decision-making could contribute toward this. Ultimately, a holistic approach combining transparency, growth prospects, flexibility, and well-being could forge a work culture that nurtures commitment, enhancing both employee retention and organisational success.

### Strengths and Limitations

This study’s strengths lie in its comprehensive examination of job retention factors among community pharmacists, relying on empirical data to identify key drivers and challenges. It notably highlights positive factors like job security, growth opportunities, and job satisfaction, offering practical insights for organisations. Additionally, it emphasises the pivotal role of management in creating a supportive work environment through care, communication, and effectiveness. This study provided valuable gender and age-based insights, aiding organisations in tailoring retention strategies. This study’s transferability across sectors is a testament to its broad applicability, making it a valuable resource for organisations seeking to foster a positive work culture, boost employee commitment, and enhancing job retention rates in the competitive job market. Its holistic approach, grounded in real-world data and practical insights, positions it as a comprehensive guide for sustainable growth and workforce empowerment. This study provides practical recommendations that are applicable beyond the pharmacy sector, emphasising transparent communication, career development, work–life balance, and managerial effectiveness, making it a valuable resource for enhancing workforce management and job retention.

This survey’s cross-sectional design hinders the possibility of establishing causal connections. Furthermore, research was carried out within a regional setting, potentially constraining the broader applicability of these outcomes. Future research could employ longitudinal designs to explore changes in job retention over time and conduct similar studies in diverse cultural and geographical settings. In this study, snowball and convenience sampling were employed to identify and recruit participants. Snowball sampling relies on referrals, potentially introducing bias as initial participants can share similar characteristics. Convenience sampling selects easily accessible participants while risking bias as they may not represent the entire population. Hence, these sampling techniques may have introduced sampling bias. Consequently, their generalisability should be cautious as this study’s findings may be limited to the specific subset of individuals within the network, potentially excluding broader perspectives.

## 5. Conclusions

This study offered a comprehensive evaluation of job retention among community pharmacists in Kuala Lumpur and Selangor states in Malaysia, considering various workforce management domains and demographic characteristics. These findings emphasised the importance of communication, care, effectiveness, and career growth in fostering community pharmacists’ job retention. Management efforts when cultivating trust, valuing pharmacists’ contributions, and offering work–life balance could play a key role in enhancing their job retention and commitment. By understanding these dynamics, organisations can tailor their strategies to nurture a dedicated and motivated workforce, ultimately contributing to both employee well-being and organisational success.

## Figures and Tables

**Table 1:** Demographic characteristics of participants.

Characteristic	N	(%)
Gender		
Male	87	(27.97)
Female	224	(72.03)
Age in years		
21–30	231	(74.28)
31–40	66	(21.22)
41–50	11	(3.54)
>50	3	(0.96)
Ethnicity		
Malay	33	(10.61)
Chinese	263	(84.57)
Indian	11	(3.54)
Others	4	(1.29)
Work experience (in years)		
≤3	213	(68.49)
4–5	62	(19.94)
6–10	24	(7.72)
>10	12	(3.86)
Current pharmacy type		
Chain	250	(80.39)
Independent	61	(19.61)
Number of pharmacists in a pharmacy		
1	61	(19.61)
2–5	234	(75.24)
>5	16	(5.14)

**Table 2:** Descriptive analysis results of job retention and workforce management domains.

Domain	Median	IQR	Strongly Disagree (%)	Disagree (%)	Neutral (%)	Agree (%)	Strongly Agree (%)
Job retention	4	4–5	1.64	5.59	22.86	56.17	13.73
Workforce management domains							
Management care	3	3–4	2.06	8.04	25.47	51.96	12.48
Management communication	3	3–4	2.32	9.97	26.11	53.12	8.49
Value and trust	2	2–3	5.92	51.06	17.68	23.41	1.93
Effectiveness	3	3–5	2.19	6.75	19.55	49.97	21.54
Career development	3	3–5	3.79	11.51	33.12	42.32	9.26
Work–life balance	2	2–3	8.75	44.76	25.27	17.75	3.47

**Table 3:** Correlation results of job retention vs. workforce management domains.

	Management Care	Management Communication	Value and Trust	Effectiveness	Career Development	Work–Life Balance	Job Retention
Management care	1	-	-	-	-	-	-
Management communication	0.32	1	-	-	-	-	-
Value and trust	0.43	0.17	1	-	-	-	-
Effectiveness	0.41	0.24	0.41	1	-	-	-
Career development	0.39	0.35	0.32	0.26	1	-	-
Work–life balance	0.13	0.18	0.21	0.29	0.27	1	-
Job retention	0.29 *	0.28 *	0.61 *	0.49 *	0.42 *	0.55 *	1

* *p* < 0.05.

**Table 4:** Ordinal logistic regression results and demographic characteristics that predict job retention.

Variables	B	SE B	Wald χ^2^	*p*	OR
Gender—male	1.224	0.691	9.027	0.045 *	1.374
Age—21 to 30 years	−0.328	0.438	4.879	0.021 *	0.756
Ethnicity—Chinese	0.316	0.073	0.296	0.462	0.653
Work experience—>10 years	0.547	0.954	10.383	0.002 *	1.215
Type of pharmacy—chain	0.085	0.035	0.852	0.578	0.284
Number of pharmacists in a pharmacy—1 pharmacist	0.234	0.518	0.137	0.624	0.312

* *p* < 0.05.

## Data Availability

The data presented in this study are available on request from the corresponding author.

## Supplementary Materials

The following supporting information can be downloaded at: https://www.mdpi.com/article/10.3390/pharmacy11050163/s1.
