# Supplementary material for: Empowering Sustainable Growth and Workforce: Unveiling Challenges and Strategies for Retaining Community Pharmacists in Malaysia
Source: Pharmacy (Basel). 2023 Oct 12;11(5):163. doi: 10.3390/pharmacy11050163 (PMC10610527; doi:10.3390/pharmacy11050163)
Supplement: Supplementary file 1 [file pharmacy-11-00163-s001.zip › pharmacy-2611819-supplementary.pdf]

Section A - Demographic characteristics

1. **Gender**
☐ Male
☐ Female

2. **Age in years**
☐ 21-30
☐ 31-40
☐ 41-50
☐ > 50

3. **Ethnicity**
☐ Malay
☐ Chinese
☐ Indian
☐ Others

4. **Work experience (in years)**
☐ ≤3
☐ 3-5
☐ 6-10
☐ >10

5. **Type of pharmacy**
☐ Chain
☐ Independent

6. **Number of pharmacists in your pharmacy**
☐ 1
☐ 2-5
☐ >5

---

7. Name of pharmacy

____

8. Location of pharmacy

____

9. Mobile number

____

In the following sections B and C, kindly select the option most relevant to your experience.

Section B - Job retention

1. The company is meeting my expectations.
☐ Strongly disagree
☐ Disagree
☐ Neutral
☐ Agree
☐ Strongly agree

2. My co-workers give me feedback in a positive manner.
☐ Strongly disagree
☐ Disagree
☐ Neutral
☐ Agree
☐ Strongly agree

3. My co-workers make me feel important.
☐ Strongly disagree
☐ Disagree
☐ Neutral
☐ Agree
☐ Strongly agree

4. There is teamwork in my company.
☐ Strongly disagree
☐ Disagree
☐ Neutral
☐ Agree
☐ Strongly agree

5. I know what is expected of me in my job.
☐ Strongly disagree
☐ Disagree
☐ Neutral
☐ Agree
☐ Strongly agree

6. I have adequate resources to do my job properly.
☐ Strongly disagree
☐ Disagree
☐ Neutral
☐ Agree

☐ Strongly agree

7. I have the freedom to communicate my points.
☐ Strongly disagree
☐ Disagree
☐ Neutral
☐ Agree
☐ Strongly agree

8. My company's work environment satisfies me.
☐ Strongly disagree
☐ Disagree
☐ Neutral
☐ Agree
☐ Strongly agree

9. My company recognises my effort.
☐ Strongly disagree
☐ Disagree
☐ Neutral
☐ Agree
☐ Strongly agree

10. My company shows concern when I am sick.
☐ Strongly disagree
☐ Disagree
☐ Neutral
☐ Agree
☐ Strongly agree

Section C - Workforce management
Domain 1 – Management care

1. I regularly receive constructive performance feedback from my manager.
☐ Strongly disagree
☐ Disagree
☐ Neutral
☐ Agree
☐ Strongly agree

2. I understand how my performance is measured.
☐ Strongly disagree
☐ Disagree
☐ Neutral
☐ Agree
☐ Strongly agree

3. I think my manager cares about me as a person.
☐ Strongly disagree
☐ Disagree
☐ Neutral
☐ Agree
☐ Strongly agree

4. My manager cares about my development.
☐ Strongly disagree
☐ Disagree
☐ Neutral
☐ Agree
☐ Strongly agree

5. My manager relieve me from work on time.
☐ Strongly disagree
☐ Disagree
☐ Neutral
☐ Agree
☐ Strongly agree

Domain 2 – Management communication

6. My manager clearly communicates expectations.
☐ Strongly disagree
☐ Disagree
☐ Neutral
☐ Agree

☐ Strongly agree

7. My manager effectively communicates the information that I need to understand.
☐ Strongly disagree
☐ Disagree
☐ Neutral
☐ Agree
☐ Strongly agree

8. My manager explains the reasons behind the decisions made.
☐ Strongly disagree
☐ Disagree
☐ Neutral
☐ Agree
☐ Strongly agree

9. My manager handles disagreements professionally.
☐ Strongly disagree
☐ Disagree
☐ Neutral
☐ Agree
☐ Strongly agree

10. My manager explains how the organisation's future will impact me.
☐ Strongly disagree
☐ Disagree
☐ Neutral
☐ Agree
☐ Strongly agree

Domain 3 – Value and trust

11. My manager creates a trusting environment.
☐ Strongly disagree
☐ Disagree
☐ Neutral
☐ Agree
☐ Strongly agree

12. My manager values everyone in the team.
☐ Strongly disagree
☐ Disagree
☐ Neutral
☐ Agree

☐ Strongly agree

13. My manager values my ideas and opinions.
☐ Strongly disagree
☐ Disagree
☐ Neutral
☐ Agree
☐ Strongly agree

14. My manager trusts me.
☐ Strongly disagree
☐ Disagree
☐ Neutral
☐ Agree
☐ Strongly agree

15. My manager values me.
☐ Strongly disagree
☐ Disagree
☐ Neutral
☐ Agree
☐ Strongly agree

Domain 4 - Effectiveness
16. I am confident in the effectiveness of my company's manager.
☐ Strongly disagree
☐ Disagree
☐ Neutral
☐ Agree
☐ Strongly agree

17. My manager has the expertise in my field.
☐ Strongly disagree
☐ Disagree
☐ Neutral
☐ Agree
☐ Strongly agree

18. My manager has the ability to help me.
☐ Strongly disagree
☐ Disagree
☐ Neutral
☐ Agree
☐ Strongly agree

19. My manager has the ability to lead my team.
☐ Strongly disagree
☐ Disagree
☐ Neutral
☐ Agree
☐ Strongly agree

20. My manager is productive in his/her work.
☐ Strongly disagree
☐ Disagree
☐ Neutral
☐ Agree
☐ Strongly agree

Domain 5 – Career development

21. My manager discusses with me career growth within my organisation.
☐ Strongly disagree
☐ Disagree
☐ Neutral
☐ Agree
☐ Strongly agree

22. My manager guides me in my career growth.
☐ Strongly disagree
☐ Disagree
☐ Neutral
☐ Agree
☐ Strongly agree

23. My manager shows interest in my development.
☐ Strongly disagree
☐ Disagree
☐ Neutral
☐ Agree
☐ Strongly agree

24. I think my manager may recommend my promotion.
☐ Strongly disagree
☐ Disagree
☐ Neutral
☐ Agree
☐ Strongly agree

25. My manager promotes those who are eligible.
☐ Strongly disagree
☐ Disagree
☐ Neutral
☐ Agree
☐ Strongly agree

Domain 6 – Work-life balance

26. My company recognises the importance of my personal life.
☐ Strongly disagree
☐ Disagree
☐ Neutral
☐ Agree
☐ Strongly agree

27. My company allow me to leave on time after working hours.
☐ Strongly disagree
☐ Disagree
☐ Neutral
☐ Agree
☐ Strongly agree

28. My company allows me to apply for annual leave.
☐ Strongly disagree
☐ Disagree
☐ Neutral
☐ Agree
☐ Strongly agree

29. My manager increases my responsibilities at work beyond my limit.
☐ Strongly disagree
☐ Disagree
☐ Neutral
☐ Agree
☐ Strongly agree

30. My manager shows concern about my family commitments.
☐ Strongly disagree
☐ Disagree
☐ Neutral
☐ Agree
☐ Strongly agree
